# Automated thigh muscle segmentation using foundation models: validation and impact of segmentation quality

**DOI:** 10.1186/s41747-026-00822-y

**Published:** 2026-10-13

**Authors:** Katharina Ziegeler, Gabrielle Hoyer, Felix Liu, Virginie Kreutzinger, Zehra Akkaya, Gabby B. Joseph, John A. Lynch, Valentina Pedoia, Sharmila Majumdar, Thomas M. Link

**Affiliations:** 1 Rheumazentrum Ruhrgebiet Herne, Ruhr Universität Bochum, Bochum, Germany; 2 Department of Radiology and Biomedical Imaging, University of California, San Francisco, CA USA; 3 Department of Bioengineering, University of California, Berkeley, CA USA; 4 Department of Bioengineering and Therapeutic Sciences, University of California, San Francisco, CA USA; 5 Department of Radiology, Faculty of Medicine, Ibni Sina Hospital, Ankara University, Ankara, Turkey; 6 Bay Area Institute of Computation, Altos Labs, Redwood City, CA USA

**Keywords:** Demographic bias, MRI, Quality assurance, Segmentation, Thigh muscle

## Abstract

**Objectives:**

This study aimed to validate a segmentation pipeline for the extraction of volumetric thigh muscle data, investigate the influence of demographics on segmentation performance, and investigate the influence of low-quality segmentations on quantitative performance and downstream metrics.

**Materials and methods:**

Manual thigh muscle segmentations on axial T1-weighted spin echo thigh images of 364 (106 training and validation; 258 testing) participants of the Osteoarthritis Initiative (OAI) were used. A trained YOLO (You Only Look Once) object detection model generated labels and box predictions, that were passed to a fine-tuned foundation model (Segment Anything, Meta), which generated muscle segmentations. The quality of segmentations was assessed by two radiologists (none, minor, major issue), and Dice scores and mean bias *versus* manual segmentations were calculated in the testing dataset. Linear regression was used to calculate the associations of age, sex, and BMI with Dice scores.

**Results:**

Dice scores ranged between 0.97 (quadriceps) and 0.89 (adductors). Exclusion of erroneous segmentations decreased mean bias by 0.06 mL across muscle groups; mean Dice of segmentations with *versus* without major issues was 0.90 *versus* 0.94. Female sex was associated with marginally lower Dice scores (beta -0.01, 95% CI: -0.02, -0.01, *p* < 0.001), while no other demographic feature showed significant associations.

**Conclusion:**

Foundation models can be fine-tuned on a modest task-specific dataset to achieve segmentation accuracy comparable to bespoke networks, without training a dedicated architecture from scratch. Visually erroneous segmentations introduce only negligible degradation of quantitative performance and downstream metrics and may thus be challenging to identify by data-driven detection strategies.

**Key Points:**

***Question*** Foundation models can be efficiently leveraged for robust segmentation on complex thigh muscle anatomy

***Findings*** Radiologist-identified erroneous segmentations introduced only negligible degradation of summary quantitative performance and are thus difficult to identify using statistical methods.

***Relevance statement*** Human-in-the-loop quality assurance post-validation adds limited value to improve downstream volumetric muscle metrics in foundation-model-based thigh segmentation. These findings carry implications for both research and potential clinical applications of muscle volume quantification, *e.g*., personalized therapeutic regimens.

**Graphical Abstract:**

**Figure:**
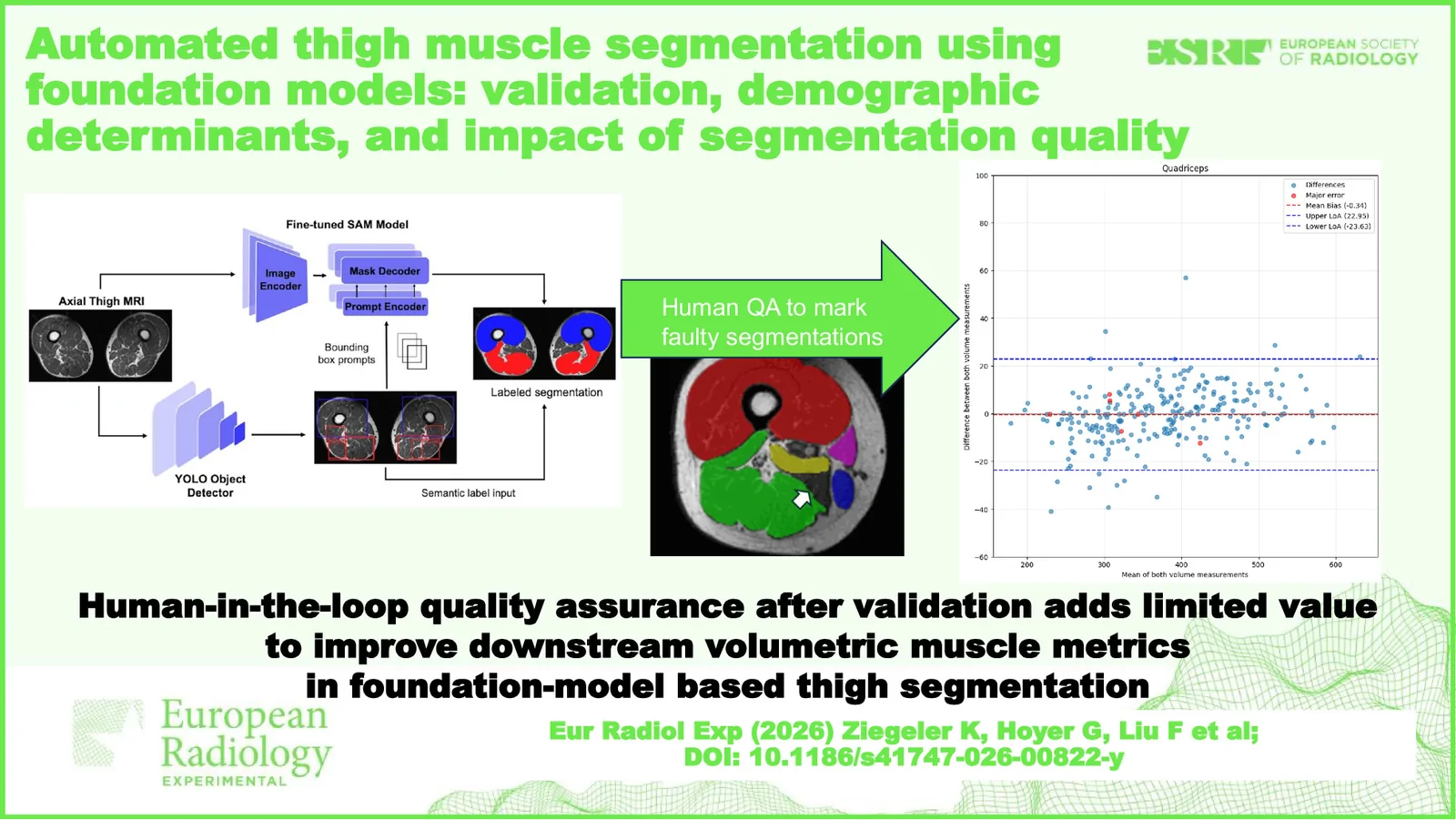

## Background

Osteoarthritis (OA) of the knee is the leading cause of disability worldwide, with a global age-standardized prevalence of knee OA of approximately 4376 per 100,000 in 2019 and a particularly high burden across Europe and the Americas [1], and an estimated national cost of $45 billion annually in the United States [2]. Despite this undisputed importance, therapeutic advances in the treatment of OA are at best incremental, and medications that have a proven effect on slowing the disease are still missing. Among the treatment interventions with the best evidence for disease modulation are those targeting muscle strength [3]. Conversely, muscle weakness has been established as a predictor of radiographic knee OA incidence [4] and progression [5]. Thus, objective assessment of muscle quality is an essential tool for both the understanding of mechanisms of disease progression, prediction of clinical outcomes, and potentially assessing the success of treatment strategies targeted at building muscle or preventing sarcopenia.

Currently applied quantitative imaging applications typically use cross-sectional area, as well as textural markers [6, 7]. A possible limitation of using a single cross-section in longitudinal assessments is the higher potential for sampling error [8]. Volumetric approaches to thigh muscle analysis therefore hold potential for more robust longitudinal assessments but have so far been technically demanding to implement, particularly because the delineation of individual functional muscle groups—which share borders with limited contrast between them—is substantially harder than segmenting the whole muscle compartment. This is reflected in reported accuracies, which fall from ~0.96 for whole-thigh muscle [9] to ~0.83–0.91 for individual muscles or muscle groups [10–12]. A further technical challenge is that bespoke networks trained for a specific task often struggle to generalize to datasets from other vendors, sequences, or biological distributions [13, 14], and conventionally require on the order of one to several hundred manually annotated scans to train [9, 15]. A promising technological advancement in anatomical segmentation is the introduction of foundation models, such as the Segment Anything Model (SAM) [16], which are pre-trained on millions of natural images. It should be noted, however, that SAM does not perform reliably on muscle MRI out of the box: its promptable, general-purpose design does not transfer directly to this domain, and reaching task-specific accuracy still requires fine-tuning on domain-specific data [17, 18]. The relevant advantage is therefore not zero-shot performance, but that competitive segmentation accuracy can be achieved by fine-tuning a pre-trained model on a comparatively modest, task-specific dataset, rather than training a dedicated architecture from scratch [17, 18].

An important caveat to fully automated approaches in digital medicine is the possibility of introducing or propagating biases from demographic factors such as sex, age, or race [19]. Examples have been found in diagnostic studies on automated diagnosis of chest radiographs [20] and detection of OA in knee radiographs [21]. Crucially, demographic bias is not confined to diagnostic tasks: deep-learning–based image segmentation itself has been shown to perform unequally across demographic groups, with significant racial disparities in Dice similarity coefficient (Dice) scores reported for cardiac MRI segmentation [22] and demographic performance gaps observed in musculoskeletal radiograph segmentation [23]. However, far less is known about the influence of demographic factors on the segmentation accuracy of skeletal muscle specifically. As the use of segmentation-derived biomarkers to predict medical outcomes is gaining traction in the imaging of OA [24], knowledge of possible demographic sources of bias in such segmentations is indispensable.

Thus, the aims of this study were (1) to establish and validate a segmentation pipeline for the volumetric assessment of thigh muscles on MRI, (2) to assess whether demographic factors contribute meaningfully to segmentation errors, and (3) to investigate the influence of low-quality segmentations on downstream metrics of diagnostic performance derived from segmentation masks.

## Materials and methods

### Cohort selection, eligibility, and data partitions

All subjects for this post-hoc analysis were obtained from the publicly available, de-identified Osteoarthritis Initiative Database (OAI; clinicaltrials.gov identifier: NCT00080171). As part of this longitudinal, multi-center observational study including individuals at risk of, or with OA of the knee, a subset of participants underwent 3.0-Tesla magnetic resonance imaging (MRI) of bilateral thighs. We used age, sex, race, and body mass index (BMI) at baseline. We included OAI baseline thigh MRI with available manual segmentations and excluded scans with incomplete masks or incomplete sequences. Table 1 details the demographic characteristics and sample size of the utilized datasets (subject level). The training and validation dataset included 106 participants with hand-segmented major thigh muscle groups, split into 80% training and 20% validation. The testing dataset comprised 258 baseline scans with ground-truth segmentations derived under the same OAI imaging protocol and annotation workflow as a previously published cohort description [17] and were not used for training or fine-tuning. The OAI received approval from the institutional review boards (IRBs) at all OAI clinical sites and the coordinating center. All participants provided written informed consent before study inclusion.

**Table 1:** Participant characteristics at baseline

Characteristic	Training and validation, *N* = 106	Testing, *N* = 258
Women, *n* (%)	48 (45.3)	110 (43.0)
Age, years, mean (SD)	58.9 (9.9)	60.3 (8.9)
BMI, kg/m^2^, mean (SD)	27.5 (4.3)	29.6 (3.5)
Race, *n* (%)		
White or Caucasian	93 (88.0)	217 (84.1)
Black or African American	13 (12.3)	36 (14.0)
Other	0 (0.0)	5 (1.9)

Values are mean (SD) or *n* (%). *N* is the column sample size; n is the count within a category. Training and validation participants are pooled. Counts and percentages correspond to the training-validation and testing splits used for model development and evaluation. Percentages use the column denominator and may not sum to 100 due to rounding. Race is self-reported using OAI categories—note the imbalance in race distribution regarding “other” category between both datasets

### Imaging protocol and label definitions and ground truth annotations

Axial T1-weighted series covering the mid-thighs were acquired with subjects in supine position; the volume spanned approximately 7.5 cm starting 10 cm proximal to the right femoral epiphysis [25]. Full sequence parameters and scanner specifications are provided as supplementary data (Table S1). Fifteen slices were obtained; the central seven were used as the unit of observation to limit edge-slice signal inhomogeneity. Within this window, five labels were analyzed: quadriceps, hamstrings, adductors, gracilis, and sartorius. Manual masks for these muscle groups were created in the Interactive Segmentation Tool for Medical Image Analysis (ITK-SNAP) (version 4.2) [26] by three board-certified radiologists (K.Z., V.K., and Z.A.). Initial masks were proposed by an internal tool [27] and then corrected slice by slice to match anatomy.

### Image and mask preprocessing

All MR images were preprocessed using a standardized pipeline. Images were clipped to the 1st–99th percentile of non-zero pixel intensities to reduce the influence of outliers, normalized to a range of 0–255, and resized to 1,024 × 1,024 pixels using cubic spline interpolation. Single-channel grayscale slices were replicated across three channels to meet the red, green, blue input requirement. Segmentation masks were resized using nearest-neighbor interpolation to maintain label integrity and exclude noise artifacts or irrelevant structures. No post-processing beyond nearest-neighbor mask resizing was applied.

### Detection-to-segmentation pipeline

The segmentation pipeline comprised two sequential stages: bounding box prediction via an object detection model, followed by muscle segmentation using a foundation model. A specifically trained You Only Look Once (YOLO) [28] object detection model was used to generate bounding boxes and class labels for each major muscle group from axial T1-weighted MR images. These bounding boxes were then used as spatial prompts for the SAM model, a prompt-based segmentation model capable of generalizing across diverse anatomical and imaging contexts.

**Fig. 1:**
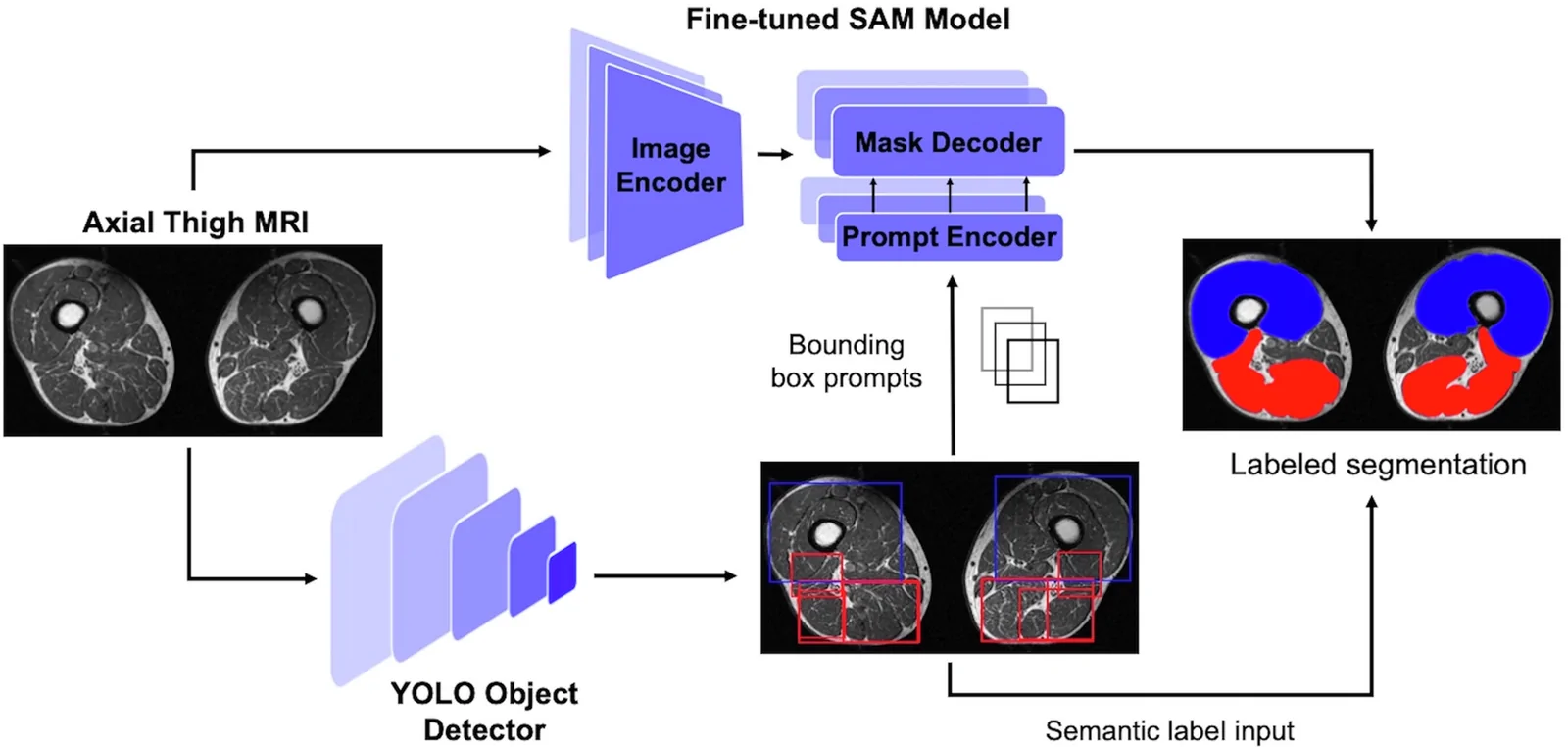
Segmentation pipeline. Axial T1-weighted thigh MR images are first processed by a YOLO-based object detector to infer muscle-specific bounding boxes and semantic muscle labels (for example, quadriceps, hamstrings), which are then passed as prompts to a fine-tuned SAM model (image encoder, prompt encoder, mask decoder) to generate labeled muscle segmentations. Example from a 73-year-old male

SAM employs a Vision Transformer (ViT-B) backbone, chosen for its balance between computational efficiency and segmentation performance. The model architecture includes three primary components: an image encoder that processes the input image, a prompt encoder that encodes the bounding box inputs, and a mask decoder that generates binary segmentation masks corresponding to the prompted region. A schematic overview of this segmentation pipeline is shown in Fig. 1.

### Training, model selection, and implementation details

SAM-ViT-B weights were initialized from the public pre-trained checkpoint and YOLO weights from the YOLOv8 public release, with random seed 42. The SAM model was fine-tuned using manual ground truth segmentations of thigh muscle groups. Mini-batch size was 2, and no augmentation was applied to bounding boxes. Optimization used the Adaptive moment estimation with weight decay optimizer (learning rate 0.0001, weight decay 0.01) with a CosineAnnealingWarmRestarts scheduler to adjust the learning rate. Gradient accumulation over four steps was used to obtain a larger effective batch size, and early stopping was applied if validation loss failed to improve after ten epochs (minimum delta 0.0001). Both the image encoder and mask decoder were jointly fine-tuned. The final model was selected as the checkpoint with the lowest validation loss. Models were implemented in Python 3.11.8 using the Python-based deep learning framework 2.3.1 (Compute Unified Device Architecture (CUDA) 11.8) and Ultralytics YOLO 8.3.11, with standard scientific Python libraries for I/O and preprocessing (NumPy, pandas, pydicom, OpenCV, scikit-image). Manual masks were edited in ITK-SNAP v4.2. Training ran on an NVIDIA Tesla V100 GPU with 32 GB memory and CUDA 11.8.

### Qualitative quality assessment protocol

**Fig. 2:**
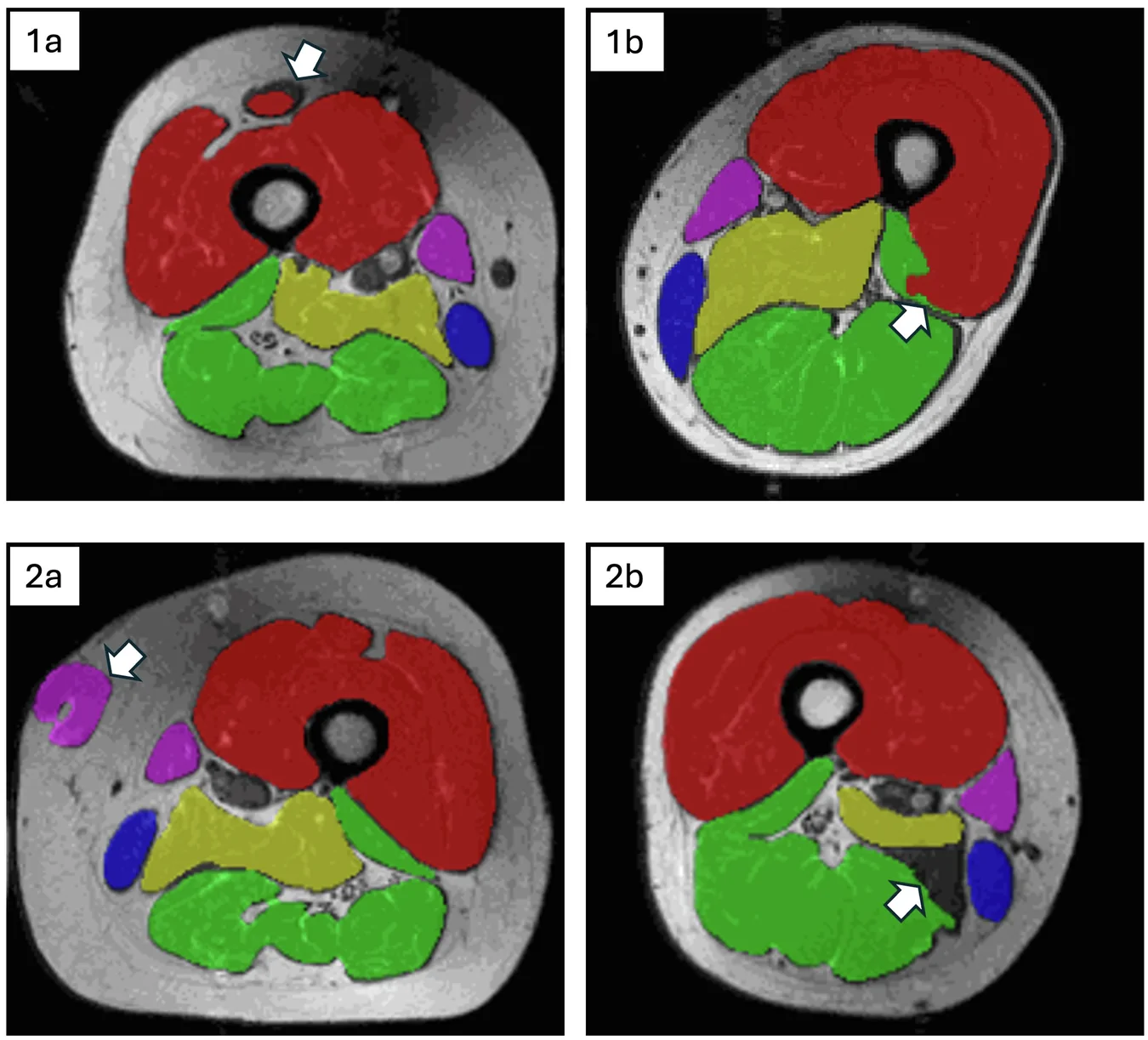
Qualitative segmentation assessment. Segmentation masks of quadriceps (red), hamstring (green), adductor (yellow), gracilis (blue), and sartorius (pink) muscles overlaid on T1-weighted spin-echo images, with errors indicated by white arrows. 1 Minor segmentation errors, with peripheral underestimation (**1a**) or overestimation (**1b**) of the quadriceps. 2 Major segmentation errors, with erroneous detection of a varicose vein as sartorius muscle (**2a**) and a larger missed portion of adductor muscle (**2b**). Demographics: **1a**, 62-year-old female; **1b**, 57-year-old male; **2a**, 59-year-old female; **2b**, 51-year-old male

Quality of segmentations was assessed using a simplified quality assurance (QA) score, adapted from Haas et al [29]: 0 = no issue, 1 = minor issue, 2 = major issue. The score was given for each label separately; examples are provided in Fig. 2. To assess intra- and inter-reader agreement for the QA score, all studies within the testing dataset were assessed by Reader 1 (K.Z., 9 years of experience) for a second time after a two-week wash-out period and by Reader 2 (V.K., 7 years of experience). Prior to scoring, a consensus reading of 20 segmentations not included in this analysis was performed by both radiologists.

### Quantitative evaluation

Technical segmentation precision was quantified using Dice scores against manual reference segmentations. Volumetric agreement between automated and manual segmentations was assessed using mean bias (automated minus manual volume, in mL and as a percentage of manual volume) and the 95% limits of agreement (mean bias *±* 1.96 times the standard deviation of the differences). For mean Dice scores and mean bias, 95% confidence intervals (CIs) were obtained via bootstrap resampling of studies (2,000 resamples).

### Statistical analysis

Dice scores are reported as mean *± *standard deviation. Differences in Dice scores between cases with no issues *versus* major issues on qualitative quality assessment were tested using two-sided *t*-tests. To assess associations between Dice scores and demographic characteristics, we fit separate linear regression models with Dice score as the dependent variable and age, sex, race (only self-identified “White or Caucasian” and “Black or African American”, due to insufficient sample size of other categories), or BMI as the predictor. To assess the influence of segmentation errors on downstream volume metrics, mean bias and limits of agreement were computed in the full test set and in the subset of cases without major segmentation errors. Furthermore, the association between radiologist-assigned QA severity and the Dice coefficient was assessed per muscle group and pooled (across groups with within-group *z*-scored Dice) using Spearman’s ρ. The ability of severity to discriminate flagged from unflagged segmentations was quantified as the area under the curve (AUC) derived from the Mann–Whitney *U*-statistic, for “no issue” *versus* “minor issue” and “no issue” *versus* “major issue” comparisons. To test whether segmentation errors could be identified automatically, per-case Dice outliers (*z*-score < -2 within each muscle group) were flagged and compared against the qualitative “major issue” cases, with detection summarized as precision and recall. To examine the laterality of segmentation performance, per-thigh Dice scores were compared between left and right sides for each muscle group using the Wilcoxon signed-rank test, and the frequency of pronounced within-subject side differences (absolute Dice difference > 0.10) was tabulated. Intra-and inter-reader agreement for qualitative ratings was assessed using weighted Cohen’s kappa (linear weights) with bootstrapped 95% CIs; values were interpreted using the scale by Landis and Koch [30]. All analyses were performed using Python. Statistical significance was set to *p* < 0.05.

## Results

### Segmentation performance

**Fig. 3:**
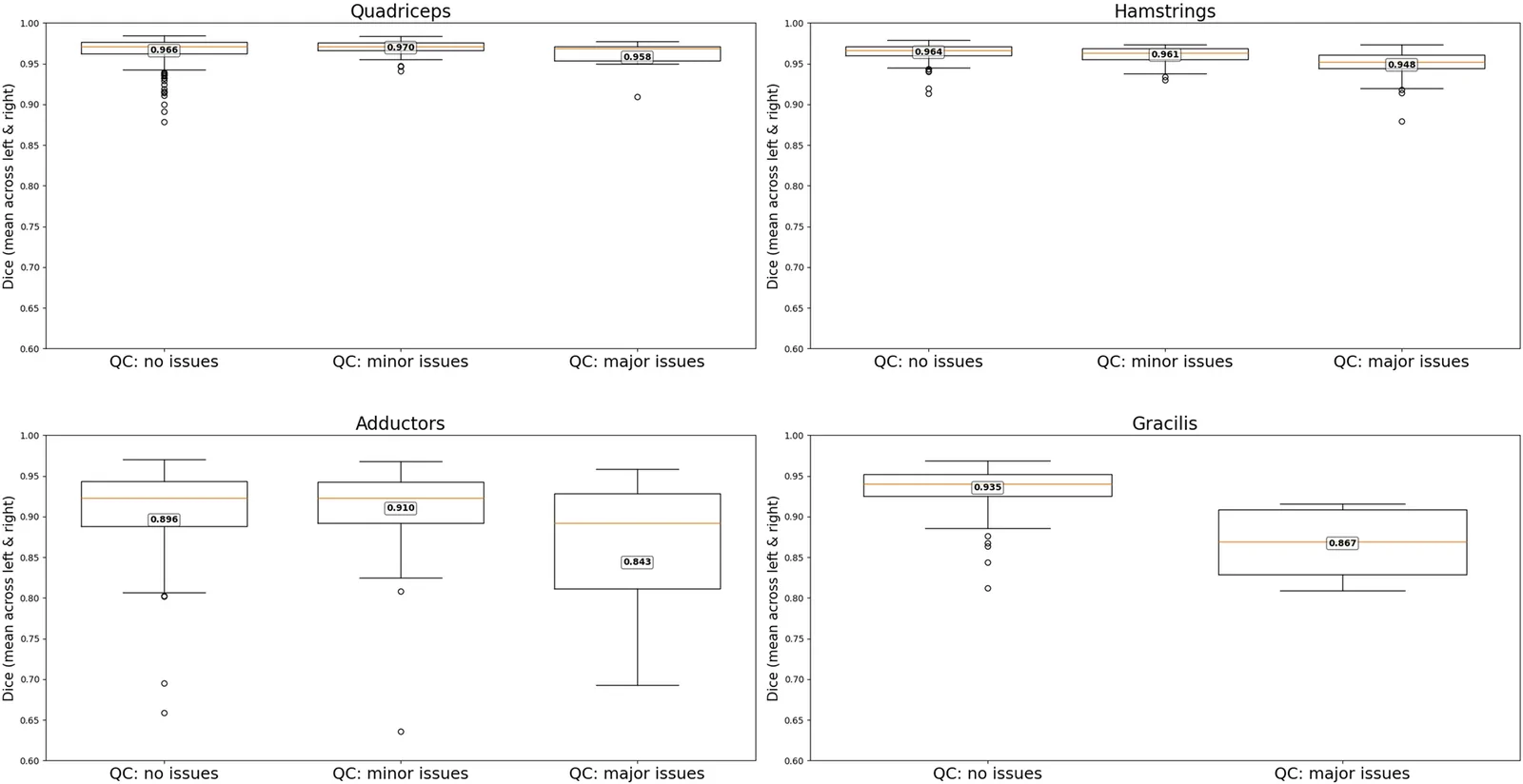
Dice scores in different subjective quality categories. Boxplots with mean Dice scores

Overall segmentation performance was excellent, with Dice scores of 0.97 ± 0.02 (95% CI: 0.96–0.97) for quadriceps, 0.96 ± 0.01 (95% CI: 0.960–0.963) for hamstrings, 0.89 ± 0.09 (95% CI: 0.88–0.90) for adductors, 0.93 ± 0.03 (95% CI: 0.93–0.94) for gracilis, and 0.94 ± 0.03 (95% CI: 0.93–0.94) for sartorius muscles. Bland–Altman plots for automatic *versus* manual segmentations are given in Fig. 3. When put in relation to the mean volume of their respective muscle groups in the ground truth segmentations, the mean bias was -0.1% for the quadriceps, 3.2% for the hamstrings, 0.2% for the adductor, 4.7% for the gracilis, and 4.0% for the sartorius muscles. Failure of segmentation during radiologists’ quality assessments was rarest in the quadriceps (2.3%; 6/258), while the highest rate was seen in the adductors (14.3%; 37/258). Only test performance is reported by design, as the validation set was used to tune the model during fine-tuning.

### Association between demographics and segmentation performance

Female sex was significantly associated with marginally lower Dice scores (beta -0.01, 95% CI: -0.02, -0.01, *p* < 0.001), while no significant association with Dice scores were found for race (White or Caucasian: -0.00, 95% CI: -0.02, 0.02, *p* = 0.948; Black or African American: -0.01, 95% CI: -0.03, 0.02, *p* = 0.664), BMI (beta 0.00, 95% CI: -0.00, 0.00, *p* = 0.823) or age (beta -0.00, 95% CI: -0.00, 0.00, *p* = 0.664). To assess confounding of sex-specific results by body composition, a multivariate model with sex, age, and BMI was calculated, which showed that the negative association with sex remained significant after adjustment (beta -0.01, 95% CI: -0.02, -0.01, *p* = 0.002).

### Quantitative *versus* qualitative performance

To investigate concordance of quantitative measures of segmentation performance and qualitative assessment by experienced radiologists, we acquired both sets of information in the testing dataset. We then compared mean Dice scores between cases without *versus* with failure detected in qualitative assessment—Fig. 3 shows boxplots of mean Dice scores per QA-score group for quadriceps and hamstring muscles. As can be seen in this figure, mean Dice scores for the quadriceps did not differ between the group of cases with no *versus* major issues in the QA, while a statistically significant but, in terms of effect size, negligible difference was found in the comparison of Dice scores of these two groups for the hamstring muscle. Results for adductors (0.90 ± 0.10 *versus* 0.84 ± 0.13, *p* = 0.007), gracilis (0.94 ± 0.02 *versus* 0.87 ± 0.05, *p* < 0.001) and sartorius (0.94 ± 0.03 *versus* 0.90 ± 0.04, *p* = 0.023) were similar to those for the hamstrings, with significant, albeit very small differences in Dice scores between segmentations without *versus* with major issues detected during QA. QA severity was only weakly associated with segmentation overlap overall (pooled Spearman ρ = −0.16, *p* < 0.001). Discrimination was strong for the most severe failures (“no issue” *versus* “major issue” AUC 0.62–0.94 across muscle groups) but at or near chance for the finer gradation (“no issue” *versus* “minor issue” AUC 0.47–0.61; non-significant for quadriceps and adductors); in the quadriceps, minor-flagged cases had marginally higher mean Dice than unflagged cases (0.970 *versus* 0.966). A simple Dice-based outlier rule (z < −2) detected QA major-issue cases poorly: recall was 0.17, 0.12 and 0.14 for the quadriceps, hamstrings and adductors, respectively (0.57 for the gracilis, *n* = 7), with precision ≤ 0.50 in all groups. Mean Dice differences between left and right thighs were statistically significant for the quadriceps, hamstrings, gracilis, and sartorius but negligible in magnitude (mean differences +0.004 to +0.008; adductors −0.001, *p* = 0.05), indicating no systematic side-dependent performance difference. Pronounced within-subject discrepancies (absolute difference > 0.10) were nonetheless present in a minority of cases and were concentrated in the adductors (30/254, maximum 0.82), with isolated occurrences in the gracilis (6/258) and sartorius (11/258) and none in the quadriceps or hamstrings.

### Influence of segmentation quality on downstream metrics

**Fig. 4:**
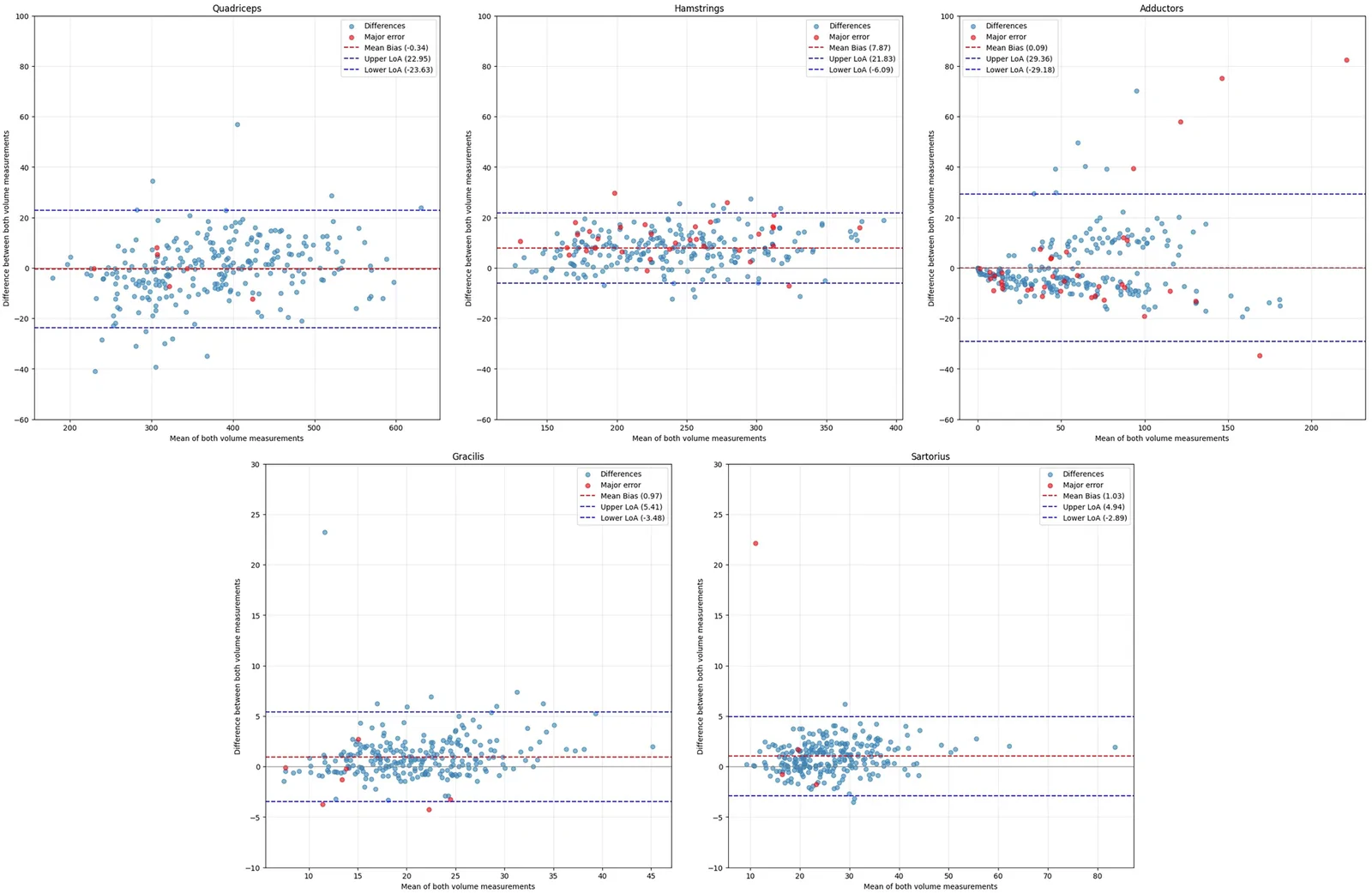
Bland–Altman plots. Plots are for volumes [mL] calculated based on automatic *versus* manual segmentation masks. Red horizontal dotted line gives mean bias; blue dotted horizontal lines indicate limits of agreement. Marked in red are cases in which automatic segmentations exhibited major errors in quality assessment. Note the adjusted *y*-axis in gracilis and sartorius, due to substantially smaller muscle size

As the outcome metric of interest, mean bias (in mL and % of muscle group volume) *versus* ground truth segmentations was chosen. In Fig. 4, cases with failed quality assessment (“major issue”) in the respective muscle groups are marked in red. As can be observed, no obvious clustering of these observations at higher distances from the mean bias was apparent. We then proceeded to investigate whether the mean bias was substantially reduced when only cases without errors in automatic segmentation masks were included. Table 2 shows absolute and relative mean bias before and after exclusion of error cases. In the full test set, bootstrap 95% CIs for mean bias were -0.34 mL (95% CI: -1.83 to 1.07 mL) for quadriceps and 7.87 mL (95% CI: 6.95–8.76 mL) for hamstrings; for all muscle groups, CIs spanned only a few milliliters around the mean. Exclusion of error cases reduced the nominal absolute mean bias for quadriceps (-0.34 to -0.17 mL), hamstrings (7.87–7.31 mL), and sartorius (1.03–0.95 mL), while for the adductors the near-zero mean bias shifted marginally from +0.09 to −0.53 mL and for the gracilis it was essentially unchanged (+0.97 to +1.03 mL). All changes were small relative to the corresponding Bland–Altman limits of agreement (*e.g*., approximately ±29 mL for adductors). Averaged across all muscle groups, exclusion improved mean bias by 0.06 mL.

**Table 2:** Mean bias and limits of agreement for automated muscle volumes *versus* the manual reference, before and after exclusion of cases with qualitative segmentation errors

Muscle group	Group	Mean bias, mL	Limits of agreement, mL		Mean bias, %	Δ |bias| , mL	Effect
			Lower	Upper		(after–before)	
Quadriceps	All	-0.34	-23.63	22.95	-0.09	-0.17	Improved
	No errors	-0.17	-24.69	24.35	-0.05		
Hamstrings	All	7.87	-6.09	21.83	3.23	-0.56	improved
	No errors	7.31	-6.53	21.14	3.15		
Adductors	All	0.09	-29.18	29.36	0.15	+0.44	worsened
	No errors	-0.53	-25.15	24.08	-0.96		
Gracilis	All	0.97	-3.48	5.41	4.75	+0.06	worsened
	No errors	1.03	-3.33	5.41	5.03		
Sartorius	All	1.03	-2.89	4.94	4.00	-0.08	improved
	No errors	0.95	-1.97	3.87	3.65		

“All” includes all testing cases; “No errors” includes only cases without qualitative segmentation issues (QA score 0). Percent bias is relative to the baseline manual volume of the same muscle group. The Δ |bias| column reports the change in the absolute mean bias after exclusion (negative = closer to the reference, *i.e*., improved; positive = further from the reference, *i.e*, worsened)

### Reproducibility of qualitative segmentation assessment

Assessments of reproducibility were performed using Cohen’s kappa with linear weights. Intra-reader reliability was almost perfect, ranging between 0.93 (95% CI: 0.87–0.97) and 0.99 (95% CI: 0.98–1.00). Inter-reader reliability was also substantial to almost perfect, ranging between 0.79 (95% CI: 0.73–0.85) and 0.88 (95% CI: 0.75–0.96); Table 3 details agreement values per muscle group.

**Table 3:** Reproducibility of segmentation quality assessments

Muscle group	Inter-reader agreement		Intra-reader agreement	
	Kappa	95% CI	Kappa	95% CI
Quadriceps	0.81	[0.71, 0.89]	0.93	[0.87, 0.97]
Hamstrings	0.82	[0.74, 0.88]	0.97	[0.94, 0.99]
Adductors	0.79	[0.73, 0.85]	0.99	[0.98, 1.00]
Gracilis	0.88	[0.75, 0.96]	0.98	[0.93, 1.00]
Sartorius	0.85	[0.71, 0.97]	0.97	[0.89, 1.00]

Weighted Cohen’s kappa (linear weights). Values shown with bootstrapped 95% CIs

## Discussion

Our proposed pipeline showed highly reliable performance for the segmentation of major muscle groups of the thigh on consecutive slices using axial T1-weighted MR imaging. Other muscle segmentation strategies, ranging from traditional convolutional architectures such as the convolutional neural network architecture for biomedical image segmentation (U-Net) [31] and nnU-Net [32] to more recent interactive foundation model pipelines like MedSAM [33] and Medical Open Network for AI (MONAI) Label [34], have shown strong potential across a variety of tasks. However, regardless of architecture, deployment of these models in either clinical or large-scale research settings requires systematic validation of outputs, particularly for anatomically complex regions like the thigh. Expert radiologist involvement remains essential to establish the nuanced limitations and strengths of segmentation models, and to evaluate their alignment with specific clinical or research goals. Our pipeline was designed to reduce the burden of expert annotation by fully automating the segmentation process while still enabling high-fidelity radiologist-led quality control. This allowed us to direct expert effort toward validating the model’s performance across a heterogeneous cohort, rather than relying on intensive expert prompting at inference. In doing so, we demonstrated a workflow better suited for scaled deployment without compromising interpretability or control over segmented data.

We specifically investigated the influence of visually low-quality segmentations on performance and downstream metrics, with a twofold aim—to assess the feasibility of deploying such a tool in scaled datasets in which rigorous quality control is not feasible, and to explore the use of downstream metrics for data-driven identification of failure cases. Although statistically significantly lower Dice scores in cases with segmentation errors in the qualitative assessment were found in all muscle groups except the quadriceps, the Dice score differences were so low (range: 0.016–0.068) that clinical relevance is questionable. Bias of volume estimations was similarly not meaningfully improved by exclusion of error cases. In fact, exclusion produced no systematic improvement in any muscle group: for the adductors and gracilis the already negligible mean bias was essentially unchanged or shifted marginally in sign, despite these groups showing the clearest QC-associated reduction in overlap (Dice 0.896 *versus* 0.843 and 0.935 *versus* 0.867 for “no” *versus* “major issue”; Fig. 3). This dissociation indicates that the visually flagged cases were not the volumetrically most deviant ones, as visual assessment and Dice capture the magnitude of overlap error, whereas mean bias reflects signed deviations that may partially offset across cases. It should be noted that this lack of influence of faulty segmentations was observed even in our sample of limited size. While these findings are reassuring for the deployment of fully automatic segmentation pipelines in a research context that focuses on group statistics, the implications for clinical care may be somewhat less optimistic. In a framework where automatically derived segmentations are used to allocate resources to individuals (*e.g*., prediction of operative success prior to knee replacement surgery), such errors as observed in our data are potentially more impactful. A simple data-driven outlier rule, however, recovered only a small fraction of qualitatively flagged errors (recall ≤ 0.17 in the three larger muscle groups) with low precision, because flagged cases rarely constituted statistical outliers and did not coincide with the volumetrically most deviant segmentations. This suggests that such errors are not readily identified by simple data-driven detection, and that quality-control strategies intended to safeguard quantitative accuracy cannot rely on overlap-based outlier detection alone.

While our assessment of segmentation quality showed robust intra- and (kappa *≥ *0.93) and inter-observed reproducibility (kappa *≥* 0.79), our results demonstrated that the segmentation errors detected by humans carry limited significance. Qualitative severity discriminated only the most severe failures (none-*versus*-major Dice AUC 0.62–0.94 across groups) but was at chance for distinguishing “minor issue” from unflagged cases (none-*versus*-minor AUC 0.47–0.61) and was only weakly associated with overlap overall (pooled ρ = -0.16, *p* < 0.001), indicating that visual grading identifies catastrophic errors but not the subtler deviations relevant to quantitative biomarkers. Given the small number of major-issue cases in the quadriceps and gracilis, the per-group estimates for these muscles should be interpreted with caution. Furthermore, in our visual quality assessment, segmentation errors were often observed on only one or two of the seven included slices, and their significance may thus have been further overestimated.

We specifically investigated, whether demographic factors, such as age, sex, race, and bodily constitution/BMI, influenced the likelihood of model failure. The only demographic feature that was significantly associated with segmentation performance was female sex. Possible explanations for this are biological in nature: as women have thicker subcutaneous fat [35] and smaller muscle volumes than men [36], the task was expected to be more challenging for the model. Reassuringly, but somewhat contrary to our initial expectation, other demographic features were not associated with weaker segmentation performance. For example, we had expected that age would decrease segmentation performance as the relative fat content of thigh muscle increases with age [37], which effectively reduces the natural contrast of muscle tissue and surrounding fatty tissue, but no significant association with segmentation performance was found.

Our study has several limitations that warrant discussion. Our quality assessment tool was developed for efficient use of human resources and general ease of use, to potentially extend to much larger data sets—we thus opted against a separate assessment of left and right thighs. As segmentation errors were often unilateral, this approach may have come at the cost of a more precise assessment of the influence of these errors. We furthermore did not test our pipeline on external, comparable datasets, in which lower segmentation performance may be expected. While muscle volume is a clinically meaningful downstream metric, it is certainly not the only biomarker that could be extracted from these imaging datasets. We did not assess any other metrics, such as intra-muscular fat [6], muscle shape, or even texture parameters, and can thus not directly infer whether these parameters show similarly low improvements from exclusion of visually faulty segmentations. These analyses remain goals for future research studies, not just in MSK radiology but also in body composition and sarcopenia research. We employed the ViT-B variant of the original SAM rather than a larger backbone (ViT-H) or the subsequently released SAM 2 [38], which became available only after our pipeline had been developed and validated; whether these would further improve segmentation performance remains to be evaluated. Relatedly, we did not systematically explore data augmentation; we aimed to characterize the baseline performance achievable by fine-tuning a foundation model on a modest dataset without extensive task-specific optimization, and augmentation represents a further opportunity to improve robustness, particularly for the more challenging muscle groups, that we did not exhaust. Although we did not benchmark against an alternative architecture, our Dice scores (0.89–0.97 across groups) are competitive with purpose-built thigh muscle-group methods, comparing favorably with 0.86 for multi-atlas/3D-deep-learning fusion of functional thigh muscle groups [11] and 0.91 for individual lower-limb muscles using a dedicated CNN [12], and approaching the 0.96 reported for whole-thigh muscle, a substantially easier, single-compartment task, on the same OAI cohort with a trained U-Net [9]. General-purpose tools such as TotalSegmentator-MRI [39] do not delineate individual thigh muscle groups and were therefore not applicable as a comparator. These comparisons are indirect, differing in cohort, sequence, and reference standard, but suggest our pipeline performs at least on par with bespoke architectures; a head-to-head comparison against a trained nnU-Net remains valuable future work.

In conclusion, our proposed pipeline showed excellent muscle volume segmentation performance, using limited training data to fine-tune a foundational model. Segmentation errors identified during visual quality control by radiologists did not introduce a meaningful bias in downstream metrics. This suggests that rigorous quality control of automatic segmentations after initial validation may not be necessary on scaled datasets in a research context, but also that data-driven approaches for detecting visually erroneous segmentations may be challenging to implement.

## Supplementary information

**Additional file 1: Table S1:** MRI imaging parameters.

## Data Availability

OAI MRI scans can be accessed via the OAI data portal (https://oai.nih.gov) with registration and a data-use agreement. Model weights are available from https://github.com/gabbieHoyer/yolo-sam-thigh-muscle.

## Abbreviations

BMI: Body mass index
CI: Confidence interval
Dice: Dice similarity coefficient
ITK: Insight segmentation and registration toolkit
ITK-SNAP: Interactive segmentation tool for medical image analysis
IRB: Institutional Review Board
MRI: Magnetic resonance imaging
MR: Magnetic resonance
OA: Osteoarthritis
OAI: Osteoarthritis initiative
QA: Quality assurance
SAM: Segment anything model
U-Net: Convolutional neural network architecture for biomedical image segmentation
ViT: Vision transformer
YOLO: You Only Look Once (object detection model)
