# Supplementary material for: Automated thigh muscle segmentation using foundation models: validation and impact of segmentation quality
Source: Eur Radiol Exp. 2026 Oct 13;10:157. doi: 10.1186/s41747-026-00822-y (PMC13661841; doi:10.1186/s41747-026-00822-y)
Supplement: Supplementary file 1 [file 41747_2026_822_MOESM1_ESM.pdf]

Automated thigh muscle segmentation using foundation models: validation and impact of segmentation quality

ELECTRONIC SUPPLEMENTARY MATERIAL

IMAGING PARAMETER	VALUE
**MRI SEQUENCE**	T1w axial
**DIMENSIONALITY**	2D
**TE / TR / SAR**	10.0 ms / 582.0 ms / 0.65
**SLICE SPACING**	5.00 mm
**PIXEL SPACING**	0.9766 mm × 0.9766 mm
**SLICE THICKNESS**	5.00 mm
**PERCENT PHASE FIELD OF VIEW**	50%
**IMAGE SIZE (ROWS X COLUMS)**	256 × 512
**FIELD STRENGTH**	2.89 T
**FLIP ANGLE**	90°
**ETL**	1

Table S1: MRI imaging parameters. Abbreviations: TE time to echo, TR repetition time, SAR specific absorption rate, ETL echo train length. Values are reported as provided in the OAI thigh sequence metadata. Scans acquired on Siemens Trio scanners, using quadrature transmit/receive knee coils (USA Instru-ments, Aurora, OH)
